# Evaluating a Web-Based Clinical Decision Support System for Language Disorders Screening in a Nursery School

**DOI:** 10.2196/jmir.3263

**Published:** 2014-05-28

**Authors:** María Luisa Martín Ruiz, Miguel Ángel Valero Duboy, Carmen Torcal Loriente, Iván Pau de la Cruz

**Affiliations:** ^1^Technical University of MadridDepartment of Telematic and Electronic EngineeringMadridSpain; ^2^Legamar SchoolMadridSpain

**Keywords:** primary health care, health information systems, knowledge management, evaluation, early diagnosis, eHealth, language disorders

## Abstract

**Background:**

Early and effective identification of developmental disorders during childhood remains a critical task for the international community. The second highest prevalence of common developmental disorders in children are language delays, which are frequently the first symptoms of a possible disorder.

**Objective:**

This paper evaluates a Web-based Clinical Decision Support System (CDSS) whose aim is to enhance the screening of language disorders at a nursery school. The common lack of early diagnosis of language disorders led us to deploy an easy-to-use CDSS in order to evaluate its accuracy in early detection of language pathologies. This CDSS can be used by pediatricians to support the screening of language disorders in primary care.

**Methods:**

This paper details the evaluation results of the “Gades” CDSS at a nursery school with 146 children, 12 educators, and 1 language therapist. The methodology embraces two consecutive phases. The first stage involves the observation of each child’s language abilities, carried out by the educators, to facilitate the evaluation of language acquisition level performed by a language therapist. Next, the same language therapist evaluates the reliability of the observed results.

**Results:**

The Gades CDSS was integrated to provide the language therapist with the required clinical information. The validation process showed a global 83.6% (122/146) success rate in language evaluation and a 7% (7/94) rate of non-accepted system decisions within the range of children from 0 to 3 years old. The system helped language therapists to identify new children with potential disorders who required further evaluation. This process will revalidate the CDSS output and allow the enhancement of early detection of language disorders in children. The system does need minor refinement, since the therapists disagreed with some questions from the CDSS knowledge base (KB) and suggested adding a few questions about speech production and pragmatic abilities. The refinement of the KB will address these issues and include the requested improvements, with the support of the experts who took part in the original KB development.

**Conclusions:**

This research demonstrated the benefit of a Web-based CDSS to monitor children’s neurodevelopment via the early detection of language delays at a nursery school. Current next steps focus on the design of a model that includes pseudo auto-learning capacity, supervised by experts.

##  Introduction

The early detection of neurodevelopmental disorders in childhood is a key task to support diagnosis and treatment processes [1,2]. The substantial role of language development, from zero until the age of 6 years, strongly influences communication and social skills in children and adults [3,4]. Furthermore, experience shows that language acquisition delays do influence social and behavioral attitudes, lack of school readiness [5], school exclusion [6], future academic problems [7], neuropsychiatric disorders [8], and poor employment [9]. Although diverse medical procedures aim to support the detection of neurological disorders in children [2,10-12], there is a lack of adoption at the primary pediatric care level, as it requires too much time and specialized knowledge [13].

Estimates of disability predominance in childhood vary due to differences in definition and the wide range of methodologies and existing measuring instruments [14]. The World Health Organization (WHO) and the World Bank declare, in their report, “World report on disability”, that many cases of children with disability are still not identified and do not receive diagnosis or treatment services from health care entities [14]. Hence, early and effective identification of children with developmental disorders remains a critical task for the international community. Language disorders are frequently the first symptoms of a possible developmental disorder [15]. The prevalence of language delays is the second highest within common developmental disorders (1-19%) [16] and it is often associated with negative long-term outcomes [3,17-19].

The mental health, social, and behavioral developmental needs of very young children have gained awareness in the last 10 years [1,20,21]. Moreover, the acquisition of communication skills is essential for all students due to its direct impact on school success [22]. Thus, the early detection of developmental disorders in early childhood may facilitate the necessary diagnosis and/or treatment actions [13], as well as the early adoption of educational recommendations and activities for professionals and parents.

Most children achieve good verbal communication by the age of 3 years [3,23], although language acquisition level has a variable range within a target population. Hence, the availability of an effective language development CDSS may facilitate early identification of these types of disorders before the age of 3 years. Both primary care and education professionals can play a valuable role in early detection during their regular interactions with a child. Unfortunately, the lack of resources to perform individualized exhaustive evaluations of all children makes the use of efficient and reliable methods of detection necessary [4]. So far as this is concerned, diverse studies demonstrate that teachers can identify pupils with language difficulties, with sufficient precision and sensibility, if they have been provided with a guide or suitable orientation [24,25].

Ygual et al discovered a significant correlation between teachers’ observations and criteria scores on intelligibility, literal understanding of sentences, grammatical expression, and lexical richness [4]. The research published by Wilson et al [3] reinforced the argument that early interventions can affect long-term outcomes, and concluded that language delay is not easily predictable from available risk factors. Therefore, it is not possible to foresee whether a child will have a language delay at 30 months and the identification of this disorder would require direct clinical contact with all families [3]. The evolution of the effectiveness of this kind of solution has been demonstrated for years, as initially explored by Miller 20 years ago [26], and surveyed by Berner and Maisiak in 1999 who concluded that a CDSS can function both to confirm and to broaden physicians’ diagnostic thinking [27].

The main objective of this research was to evaluate the deployment of a Web-based system for efficient screening of language disorders at the early stages of a child’s development. The implemented solution is a Clinical Decision Support System (CDSS), called “Gades”, whose use was widely tested in a nursery school. This paper discusses the results obtained from the Gades validation to provide professionals with real-time knowledge on early identification of 146 children with possible language disorders. The previous publication of Gades’ user requirements, implementation, deployment, and validation showed high success from a usability point of view [13].

The development of a knowledge base (KB) [28], needed to build the CDSS, relied on incremental interactions and refinement with the experts community. A set of 41 retrospective cases of children, treated over 15 years at the Language Intervention Center (LIC) of La Salle University, Madrid, helped to fine-tune the questions in the KB, starting with well-accepted neurodevelopmental tests.

## Methods

### Gades Knowledge Base

Gades KB is based on an ontology that integrates a child’s language acquisition information according to age. It has over 100 rules to generate alerts and/or alarms in case deviations from the child’s development stage are detected. The initial version of Gades KB was built between September 2011 and April 2013, according to the experience of a multidisciplinary team. Two language therapists, a neuropediatrician, a neonatologist, and three engineers supplied inputs for Gades KB, updating original versions of the Denver Developmental Screening Test. The team used CommonKADS (CK) methodology [29], to design and develop a decision support system based on the knowledge extracted from human experts and its required codification for system processing [29]. The baseline of Gades KB was Denver Test, as it is widely accepted in primary pediatric care [30]. The Gades KB takes advantage of the monthly structured questions of Denver. Each of the items of Denver represent the mean of the language development for each month of the child’s life. The first version of the KB included 136 questions, from month 1 to month 72. These questions belong to two main categories: (1) questions called “Alert Milestones” that recommend a visit to the pediatrician. A negative answer to these developmental items means that the child should make a regular follow-up visit within 3 months to re-evaluate the level of language acquisition, and (2) questions called “Alarm Milestones”, that suggest direct referral to a specialist in language disorders.

The Gades KB evaluates four areas of speech and language development: Sensory Reception (SR), Speech Perception (SP), Speech Production (SPD), and Pragmatic (P). The Gades system relies on Gades KB to support early detection of language disorders.

### Gades Clinical Decision Support Web-Based System

**Figure 1:**
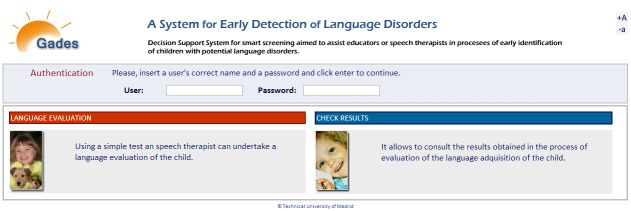
Gades home page and main functionality.

The Gades system aims to enhance early detection of children with language disorders. Its evaluation process involved 146 children attending Legamar Nursery School. The Gades KB integrates all the knowledge and logic associated with the decisions supported by the system. The potential outcome is the suggestion of early referral to specialists if a child under 6 years old may have a language disorder. Figure 1 shows the Gades Web interface whose home page includes the following functionality: user authentication, language evaluation, and results obtainment.

### Gades Deployment and Evaluation Method

A Nursery School Language Therapist (NSLT), employed at Legamar Nursery School, Madrid, evaluated the Gades system in the spring of 2013. A total of 63 boys and 83 girls participated in the study; 94 children from 0 to 3 years old and 52 children in the 4-6 years stage. The number of enrolled children in the 0-3 years group was higher since the early detection of language disorders is a research priority in this developmental period. The entire staff of educators at Legamar observed and evaluated the behavior of the children, by following the questions suggested by Gades. The average age of the 12 teachers at Legamar was 34 years old. All the educators and the NSLT were women with little background in information technology.

**Figure 2:**
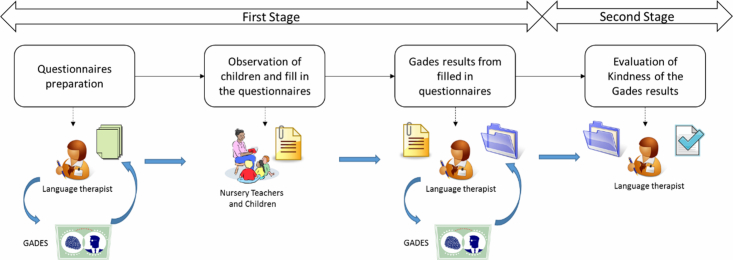
Evaluation method for early detection of language disorders.

The study started 6 months after the beginning of the school year to ensure that teachers had enough information about their pupils. Figure 2 shows the two stages of the methodology, with the same NSLT in both. The first stage involved language evaluation of all the children. The Gades KB helped the NSLT to obtain questionnaires for every group of children who participated in the study. Educators received the paper questionnaires along with an initial training session. The NSLT proposed child observation for one week before starting the evaluation process. After the observation period, educators filled out one questionnaire per child and gave them back to the NSLT. The questionnaires provided by the educators with their perceptions of the child enhanced the information acquisition process and gave the NSLT better evidence for each child enrolled in the study. The NLST updated the children’s data in Gades to avoid usability problems or system interaction barriers. In the second stage, the accuracy of the Gades results was evaluated. The NSLT validated the results for each evaluation and stated whether or not she agreed with the Gades evaluation. The NSLT also checked the questions relating to the aforementioned results. When the NSLT did not agree with the Gades decisions, she analyzed the language areas evaluated by the KB questions. Thus, the NSLT considered non-evaluated language areas and proposed modifications in order to improve the Gades KB.

### Sex and Age of the Population Enrolled in the Study

Table 1 summarizes the population and number of language evaluations carried out by age and sex. All children at Legamar participated in the study and their distribution shows a higher ratio of girls at all ages (except the age of 5 years where there were more boys). Overall, 56.8% (83/146) of evaluated children were girls and 43.2% (63/146) were boys.

In terms of age, the number of subjects involved in the study at age 3 was higher (48 children from the sample of 146 subjects), since there was a higher number of children enrolled at nursery school at this age. From a prevention point of view, the detection of language disorders before the age of 3 years is a key issue as it directly influences the Quality of Life related to Health (QoLrH). Hence, 100% of the children between 0 and 3 years at Legamar participated in the research. However, only 37.1% (52/140) of the children between 4-6 years were included, as early detection is not significant at this stage. In the 0-3 years stage, a population of 94 children participated in the study, and the distribution of children who attended the nursery school was 59% (55/94) girls and 41% (39/94) boys. In the 4-6 years stage, 52 children of the nursery school received language evaluation by Gades: 54% (28/52) were girls and 46% (24/52) were boys.

**Table 1:** Number of language evaluations conducted by age and sex.

	Year 1	Year 2	Year 3	Year 4	Year 5	Year 6	Total
Boys	5	14	20	7	14	3	63
Girls	6	21	28	14	8	6	83
Total	11	35	48	21	22	9	146

## Results

### Overview

The following section details the language evaluation results, obtained from the feedback supplied by the NSLT and teachers who participated in the research. Table 2 relates the global results extracted from the evaluation of 146 children with the Gades decision acceptance ratio, classified by developmental stages and years. The NLST accepted 122 cases resulting in an 84% success ratio. This ratio is even higher at the 0-3 years stage, where 87 cases (93%) were accepted from a total of 94. The NSLT disagreed with the Gades outcome in 24 cases (16%). The higher concentration of this non-acceptance ratio focuses on the 4-6 years stage (17/24). As previously noted, the NLST agreed with the decision in 87 cases at the 0-3 years stage and rejected Gades outcomes in 7 cases (7%). The successful cases reached a total of 35 children in the 4-6 years stage with 17 decisions requiring additional review. The total number of cases between years 5 and 6 is lower, with 9 evaluated children, since the experiment did not include children who had reached 6 years old before the spring term. The ratio of non-accepted decisions is too high at this stage (8/9), which suggests the need to improve the sample and KB for this group. The best Gades outcomes happened in the 25-36 months group where the NLST positively accepted all the suggestions (48). Table 2 provides further details with absolute and comparable relative data.

Although the NSLT agreed with all the questions for several months, she pointed out the need to refine the KB in order to improve Gades’ decisions. For example, discrepancies arose between Gades’ decision and the NSLT—the therapist recommended postponing some questions, which are not yet required for some months in the second year. At years 4, 5, and 6, the NSLT requested adding questions related to the articulation of the language and pragmatic ability. The KB refinement will require cooperation between the NSLT at Legamar and other speech therapists.

A key result obtained from the Gades evaluation was the identification of possible language delays in 7 children who had not previously caused alarm to either the NSLT or his/her educator. These cases require a formal diagnosis process in order to compare the system’s decision with traditional methods. These children had been enrolled for a few months at Legamar and the speech therapist had not noticed any delays. Table 3 summarizes the decisions provided by the NSLT after the formal evaluation.

The NSLT identified discrepancies in cases 1, 2, and 3, described in Table 3, between the behavior observed in the school environment and the behavior confirmed by parents. A typical explanation is the deviation of the linguistic functionality of some children, mediatized by the difference between the language used at the school and the one used at home by the family. The reliability of the observation, carried out by parents or relatives, always needs to be checked to avoid subjective approaches. Gades’ outcomes led to the initiation of early therapeutic actions at Legamar in cases 4 to 7.

**Table 2:** Number of language evaluations performed and therapist decisions.

Stage	Months of the questions	Year (age) of the child	Total number of cases	Number of cases where NSLT^a^ accepted Gades decision (%)	Number of cases where NSLT did not accept Gades decision (%)
0-3 years	0-12	Year 1	11	11 (100.0)	0 (0.0)
	13-24	Year 2	35	28 (80.0)	7 (20.0)^b^
	25-36	Year 3	48	48 (100.0)	0 (0.0)
	Total		94	87 (92.6)	7 (7.4)
4-6 years	37-48	Year 4	21	13 (61.9)	8 (38.1)^c^
	49-60	Year 5	22	21 (95.5)	1 (4.5)^c^
	61-72	Year 6	9	1 (11.1)	8 (88.9)^c^
	Total		52	35 (67.3)	17 (32.7)
Total			146	122 (83.6)	24 (16.4)

^a^NLST: nursery school language therapist.

^b^The age in months of some questions is incorrect. Therefore, the NSLT believed that some questions should be delayed.

^c^Incorporating additional questions related to the articulation of language and pragmatics is required.

**Table 3:** Decision of the NSLT^a^ on 7 new cases of children with possible language delays.

Case	Age and sex of the child	NSLT opinion
1	21 months – Girl	She walked at 19 months and she is very shy and inhibited. She was referred to motoric stimulation. Four month after the Gades evaluation, the observation process continues because she is still in process of adaptation.
2	18 months – Girl	She was brought to early attention. She was detected with a motor delay.
3	26 months – Girl	She had begun motoric treatment with 8 months. After Gades evaluation she started speech therapy treatment.
4	34 months – Girl	After Gades evaluation she started speech and language intervention.
5	39 months – Boy	After Gades evaluation he started speech and language intervention.
6	36 months – Boy	After Gades evaluation he started speech and language intervention.
7	42 months – Boy	After Gades evaluation he started speech and language intervention. The NSLT suggested that he is a child with family problems that may have affected the delay.

^a^NLST: nursery school language therapist.

### Knowledge Base Accuracy

**Figure 3:**
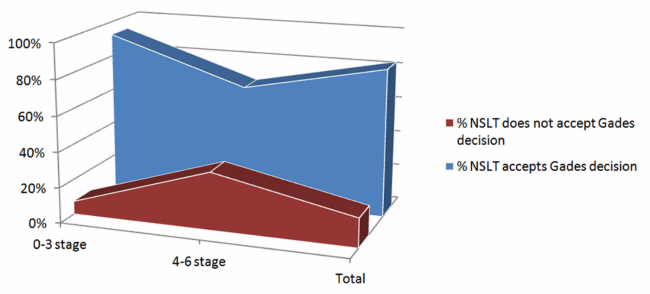
Knowledge base accuracy by stage.

The NLST accepted Gades’ decisions in 93% (87/94) of the 0-3 years cases and 67% (35/52) at the 4-6 years stage. Figure 3 shows that disagreements with Gades’ decisions are higher in the 4-6 years stage where the NSLT indicated that some of the KB questions should be reviewed. The results comparison led to a total accuracy ratio for Gades KB of 84% (122/146). A total of 24 cases from the 146 sample set a 16% non-acceptance ratio to be reduced with further KB refinement. The NLST and experts consider that a golden pattern for Gades KB accuracy of 95% will be achieved after the ongoing review of pragmatic and language articulation items.

**Figure 4:**
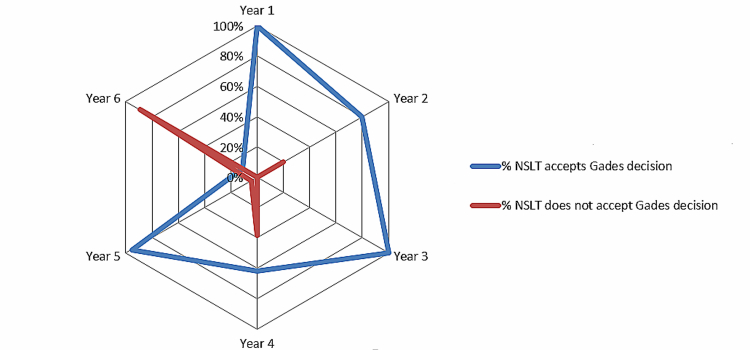
Knowledge base accuracy by age of the child.

Figure 4 compares the acceptance of Gades’ decisions in years 1 to 6. The area representing the therapist’s agreement with the GADES system is greater than the area that expressed her disagreement. Years 1 and 3 reached a 100% acceptance ratio and year 5 up to 95%. The NSLT did not accept some Gades decisions at the fourth and sixth year, due to lack of agreement with some of the KB questions. The NSLT acceptance ratio of 80% of Gades decisions at year 2 has led to a recent update of the KB to enhance the system outcomes at this stage.

The accuracy of the KB questions after the language evaluation process at Legamar is grouped by year in Table 4. The second and third columns show the age range in months and the corresponding number of evaluated questions for each age range. The KB should have a minimum and necessary set of questions. The group of experts, who participated in the KB construction, stated that a range of 3-8 questions per month may be enough to achieve early detection of language disorders. Thus, the desired maximum number of questions in this range would be 48 (8 questions per month as a maximum) and this value is not reached in any group. The current version of the KB has a small number of questions for each month.

There are more questions at the 0-3 years stage, because early detection of language disorders is critical at this developmental period. The child evolves very quickly at this stage and the KB requires higher accuracy to analyze the evolution status. There are not questions for all months in the 4-6 years stage, because the therapists determined during the process of KB construction different age ranges to support a specific assessment. Questions are structured according to evaluative items at 42, 45, 46, 48, 54, 60, 66, 69, and 72 months.

The fourth column of Table 4 indicates the language development areas, evaluated by the KB questions. Finally, the last column details the opinion of the NSLT about the evaluated areas: correct questions or questions to be added, according to the KB for each year.

The NSLT indicates that the separation between speech perception and pragmatic is minimal. Besides, pragmatic disorders often coexist with other language problems such as vocabulary development or grammar. Pragmatic problems have lower social acceptance. The NSLT considers that the correct evaluation of pragmatics is important to avoid, or to treat as early as possible, a future neurological disorder.

**Table 4:** Accuracy and refinement of the knowledge base questions.

Year of the questions	Months of the questions	Number of KB questions	Evaluated areas	NSLT^a^ opinion
Year 1	0-6	18	SR^b^ - SP^c^ - P^d^	Questions OK
	7-12	23	SP - SPD^e^ - P	Questions OK
Year 2	13-18	17	SP - SPD - P	Questions OK
	19-24	18	SP - SPD - P	Disagree with some questions
Year 3	25-30	13	SP - SPD	Add questions of P
	31-36	10	SP - SPD	Add questions of P
Year 4	37-42	5	SP - SPD - P	In SP more questions of articulation language More questions of P
	43-48	11	SP - SPD - P	Questions OK
Year 5	49-54	3	SP - SPD	In SP more questions of articulation language Add questions of P
	55-60	4	SP - SPD - P	Add questions of P
Year 6	61-66	3	SP - SPD - P	Add questions of SP and SPD
	67-72	9	SP - SPD - P	Questions OK

^a^NSLT: nursery school language therapist

^b^SR: sensory reception

^c^SP: speech perception

^d^P: pragmatic

^e^SPD: speech production

### Evaluations Percentage by Result and Sex

**Figure 5:**
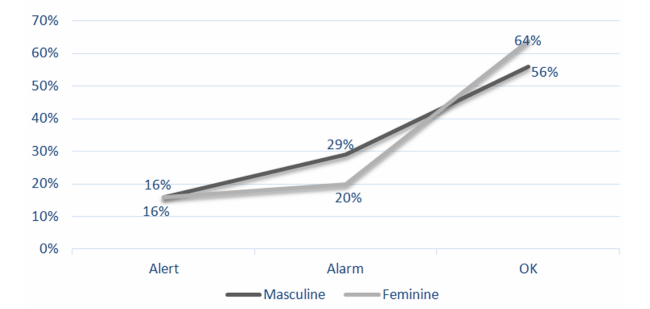
Evaluations percentage by result and sex.

Figure 5 illustrates that the percentage of alerts suggesting a pediatric visit is equal in both sexes. Despite having fewer language evaluations of boys than girls, it is remarkable to have a higher percentage of normative results (OK) for girls than for boys. Besides, most of the alarms, implying immediate referral to a specialist, occur for boys.

The language evaluation identified a total of 88 cases with a normative result (OK), a total of 35 cases with a referral to a specialist (Alarm), and a total of 23 with a follow-up pediatric visit (Alert). According to the sex of the child, a total of 83 girls were evaluated with the following results: 13 cases (16%) with Alarm, 17 cases (20%) with Alerts, and 53 (64%) cases with OK. A total of 63 boys were evaluated with the following results: 10 cases (16%) with Alarm, 18 cases (29%) with Alerts, and 35 cases (56%) with OK.

### Evaluations Percentage by Result and Age

**Figure 6:**
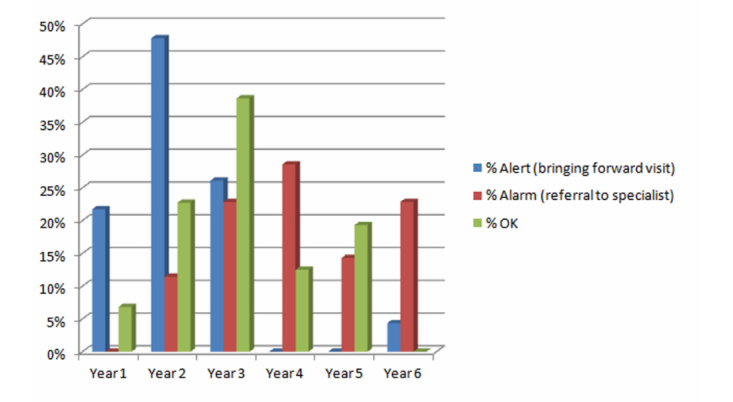
Evaluations percentage by result and age.

There were more language evaluation cases of children with normal development (OK) in the third year as Figure 6 illustrates. However, there were no abnormal language evaluations in the sixth year. A large percentage of language evaluations during the sixth year were referrals to a neuropediatrician or early attention (Alarm). This is due to the fact that there was a question that none of the children satisfactorily answered, which justifies the need to refine the KB before conducting new language evaluations with children or evaluations in the primary care real environment.

The maximum number of language evaluation cases of children with an Alert happened in the second year. The percentage of Alerts in years 1, 2, and 3 is higher than in years 4, 5, and 6. The higher percentage of Alarms at the 4-6 years stage is not significant because the NSLT detected some semantic mistakes in some KB questions. The current refinement of the KB is taking into account the opinion of the group of experts who originally participated in the KB construction and the evaluation results of Gades presented in this work. For this reason, the KB questions will not be reviewed until they can include the review from all experts, according to the analysis of the results of the Gades evaluation process summarized in Tables 2 and 3. After this, the modifications suggested for the KB refinement process will be adopted and a second evaluation process will be triggered with two more enrolled schools. Thus, some enhancements are expected in the NSLT acceptance of Gades according to the suggested refinement proposed to the system for years 4, 5, and 6.

## Discussion

### Principal Findings

Although previous studies relate positive experiences about the educator role in the process of language acquisition [4,31-38], the real impact of CDSS needs a deeper discussion. The adequate formalization of knowledge acquisition about associated symptoms in certain contexts conditions the reliability of Gades ontology, created for early detection of language disorders. The results obtained in this research, acquired from the input of 12 educators at the Legamar school, are consistent with the approach of Ygual-Fernandez et al, which supports feedback from nursery school educators to validate the decisions, as triggered by Gades. System outcomes validated in this research do align with recent studies like [39], which reinforce the use of computers, handheld, and mobile devices to provide instant access to extensive amounts and types of suitable information for health care professionals.

Language evaluation performed by Gades is consistent with the higher incidence of language impairment between boys and girls identified in scientific studies [3,40]. This research did not start from an equal ratio of male and female populations because it assumed the unbalanced gender distribution of the whole population of the targeted nursery school. However, the size of the sample (146 children) provided Gades with the capacity to support a significant aggregation of the results for both age and sex. The continuity of the training period, carried out for 3 months by the speech therapist with the educators, positively influenced their acceptance of Gades procedures and observations. This step-by-step methodological approach helped to foster the results obtained, which were ultimately able to successfully indicate the existence of language disorders in children.

The higher accuracy of Gades at 0-3 years than at the 4-6 years stage is directly related to the research rationale that gave priority to earlier and more precise detection of language disorders from 1 to 3 years old. This outcome does not invalidate the use of the system from 4 to 6 years old but paves the way for better performance at this stage after the KB refinement. Furthermore, the comparison between Gades’ results and the expert’s feedback shows that linguistic functionality from 30 months cannot be clearly formalized though a specific item. The NSLT pointed out the need to enhance the analysis of pragmatic skills at each contextual scenario from 3 years onward. The deeper specific review of Gades KB, including more specific questions of pragmatic evaluation, will require a second wide scale evaluation to measure the improvements of effectiveness and reliability for language evaluation results in the 4-6 years stage.

The definition of two incremental phases in the evaluation method of Gades’ capacity for early detection of language disorders helped to provide users with requested information packages at the stage of need. The design of traditional questionnaires for parents and teachers, adapted from Gades KB, made it easier to assess the language use of the child in different interaction environments [32]. For example, the information offered by parents and teachers through the Children’s Communication Checklist (CCC) demonstrated good sensibility and determined pragmatic difficulties that children might present such as autism, attention-deficit/hyperactivity disorder (ADHD), conduct disorder, Williams’s syndrome, and Down’s syndrome [4].

### Limitations

The early detection of language disorder tools has well-documented limitations in the specialized literature [4], such as: (1) subjectivity of the person who completes the questionnaire or scale of values [33], and his/her previous knowledge or specific training in relation to linguistic skills; (2) inconsistencies between the teacher’s observations and the child’s capacity in evaluation tests [34,35], due to possible differences in the child’s linguistic conduct in spontaneous daily situations and to his/her execution during a formal evaluation, characterized by a major inflexibility [36]; and (3) trustworthiness of the predictive power of the questionnaires used due to the fact that they depend on the age of the children, where the estimations of teachers seem to be less trustworthy when smaller children are evaluated, in relation to the rapid cognitive and behavioral changes that they try out in these early ages [37,38].

The limitations of the aforementioned issues do not invalidate the current research as a high number of studies verified the existence of a significant correlation between the observations of teachers with diverse linguistic skills and the punctuations obtained by his/her pupils in different standardized evaluation tests. All of them used questionnaires focused on general or punctual aspects of linguistic processing as Gades inputs did [4]. Other works have also reported teacher difficulty in the detection of speech difficulties and a lack of sensibility for the differentiation of difficulties in the speech domain in every evolutionary moment [32].

Although the Legamar school is a private entity, its demographic data show a realistic potential to scale up the trials and results of this research to other public and private centers. It assists children from middle-class families with a normal distribution of gender, age, and parental income. The extrapolation of the study to other classes in public or private schools does not require methodological changes or a team of professionals to be involved. A higher number of children with language delays is expected to be obtained in a center where speech therapists do not belong to the regular staff. If the school does not have an NSLT, the method presented in this study cannot be applied equally and the NSLT functionality could fall to educators or another professionals.

Consulted language experts stated that the extrapolation of the study to another region where other dialects or languages are spoken may obtain similar results. Children all over the world learn more than one language without developing speech or language problems. Even though bilingual children develop language skills just as other children do [41], the introduction of a second language may slightly delay the acquisition timescale. This "silent period" can sometimes last several months. This is a normal evolution and the child will recover the proper developing stage [41].

Finally, we have not detected false negatives in any stage (0-3 or 4-6 years). The false positive rate in stage 0-3 years has been low. However, we had a high ratio of false positives in the 4-6 years stage. The NSLT detected the main causes of this ratio to be related to semantic mistakes in the questions involved. We are currently in a refinement process to solve this situation.

### Conclusions

This research details an innovative solution to support knowledge-based detection of language disorders in children aged 0 to 6 years at nursery schools. The solution provides nursery school educators with a monitoring tool to assess the degree of language acquisition in their students. In spite of the additional workload faced by the educators, the school highlights the benefits of this type of monitoring for children.

The results of the evaluation at the Legamar Nursery School show that several children identified by Gades as having a possible language delay had not previously caused alarm to either the school therapist or to his/her educator. Further, a large number of children identified by Gades were also identified by the NSTL, especially in the 0-3 years stage. These results lead us to conclude that this kind of Web-based CDSS can undertake early detection of language delays in children at a nursery school with the support of their teachers, thus improving the neurodevelopmental follow-up.

In the process of early detection of language disorders, it is necessary to have not only a very specific knowledge, but also, a capacity for suitable observation. Therefore, we can summarize that Gades can be an effective CDSS for use by speech therapists and school psychologists in the rapid detection of children with difficulties in language development; Gades guides educators in the observation required for detection and also promotes the stimulation of skills aimed at diminishing and even preventing the appearance of these disorders; and Gades can be a collaboration tool involving parents and primary care pediatricians in the process of language evaluation.

Other conclusions of this research suggest the need to include supervised learning capacities in Gades. The learning functionality requires the definition of a specific model that allows a proper mix of automation and experts’ supervision. Experts will be able to update Gades KB easily, taking into account the suggestions triggered automatically by the system. These suggestions will come from significant samples of real use cases. The following complementary proposed actions will improve the capacity of Gades detection in order to promote better health status of children. First, questions related to difficulties in the sound articulation domain can be incorporated. There can be situations in which children do not have a problem in language development, rather the problem derives from difficulties with sound discrimination. These questions will be studied by a multidisciplinary team of experts skilled in the relevant areas. Second, complementary evaluation in other areas outside of the school can be included. The observation capacity of the teachers, though it is considerable, does not include all aspects that would be desirable at the time of establishing a proper diagnosis. To be able to analyze other contexts outside of school, there is a version being adapted for primary care pediatricians and analysis has also begun for a possible version for parents. Third, new questions to improve aspects in the language domain can be refined and added. In the 4-6 years stage, the need for major refinement was detected in questions related to the pragmatics. Currently, we are working on it with the NSLT at Legamar.

Furthermore, the authors have defined a new concept called “Internet of Toys”. It deals with the possibility of obtaining information about the child’s development through his or her natural interaction with toys. This new interaction paradigm might provide Gades with the capacity to acquire real-time data in order to improve its reasoning performance. Thus, the system could improve its effectiveness thanks to the very earliest utilization of information related to the behavior of the child. Data monitored via the expected interaction of children with certain toys could enhance Gades’ reliability with more critical information.

## Abbreviations

ADHD: Attention-Deficit/Hyperactivity Disorder
CCC: Children's Communication Checklist
CDSS: clinical decision support system
CK: CommonKADS
KB: knowledge base
LIC: Language Intervention Centre
NSLT: nursery school language therapist
P: pragmatic
QoLrH: Quality of Life related to Health (QoLrH)
SP: speech perception
SPD: speech production
SR: sensory reception
WHO: World Health Organization
